# Primary immature teratoma in the liver with growing teratoma syndrome and gliomatosis peritonei: a rare case report

**DOI:** 10.1186/s13000-022-01267-8

**Published:** 2022-10-29

**Authors:** RenMing Liu, JianNing Chen, ChunKui Shao, Na Cheng

**Affiliations:** Department of Pathology, The Third Affiliated Hospital, Sun Yat-Sen University, No. 600 Tianhe Road, Guangzhou, 510630 People’s Republic of China

**Keywords:** Immature teratoma, Liver, Growing teratoma syndrome, Gliomatosis peritonei

## Abstract

**Background:**

Primary liver immature teratoma is extremely rare and only 4 cases have been reported, let alone with growing teratoma syndrome (GTS) and/or gliomatosis peritonei (GP).

**Case presentation:**

Here, we report a case of a 44-year-old female presenting with progressive abdominal distension and elevated serum alpha fetal protein (AFP) level. CT/MRI scans revealed a large cystic-solid mass in the right lobe of the liver, accompanied with implant or metastasis in the abdominal cavity. Pathologic examination at biopsy suggested immature teratoma. After 4 cycles of chemotherapy, an MRI showed a slight increase in tumor size. Therefore, surgical resection of the right lobe of the liver was performed. The final histological diagnosis was a mature teratoma (tumor size 28 cm × 14 cm × 13 cm), with no residual immature component, and the diagnosis of GTS was considered. The patient continued to receive 2 courses of postoperative chemotherapy. An abdominal CT scan revealed innumerable miliary nodules in bilateral adnexal areas 2 months after surgery. Histologically, large numbers of mature glia were observed, supporting the diagnosis of GP.

**Conclusions:**

We report for the first time a case of primary liver immature teratoma with GTS and GP in an adult. Longer follow-up is needed to assess definitive efficacy.

## Background

Teratomas are germ cell tumors composed of multiple tissues derived from two or three germ layers (ectoderm, mesoderm, and/or endoderm). According to whether they contain immature tissues (typically primitive/embryonal neuroectodermal tissues), teratomas can be divided into mature and immature subtypes [1, 2]. The majority of teratomas originate from gonads, while the others occur in extragonadal parenchymal organs, such as bladder, prostate and even liver [3–7]. Alpha fetal protein (AFP) level in patients with immature teratoma is usually elevated markedly. If serum AFP level returns to normal after systemic chemotherapy but the tumor continues to increase in size, a diagnosis of growing teratoma syndrome (GTS) should be considered [8–11]. And when mature glial tissue implants widely on peritoneum, it is called gliomatosis peritonei (GP) [12, 13]. Primary liver teratoma in adults is exceedingly rare, with only 14 cases reported in the literature, 10 of which were mature teratomas [14–19] while the other 4 cases were immature teratomas [20–23]. Herein, we first report a case of adult primary liver immature teratoma with GTS and GP, with no relevant literature has been reported, aiming to increase awareness of this rare disease, further optimizing the treatment strategy and improving overall prognosis.

## Case presentation

**Fig. 1:**
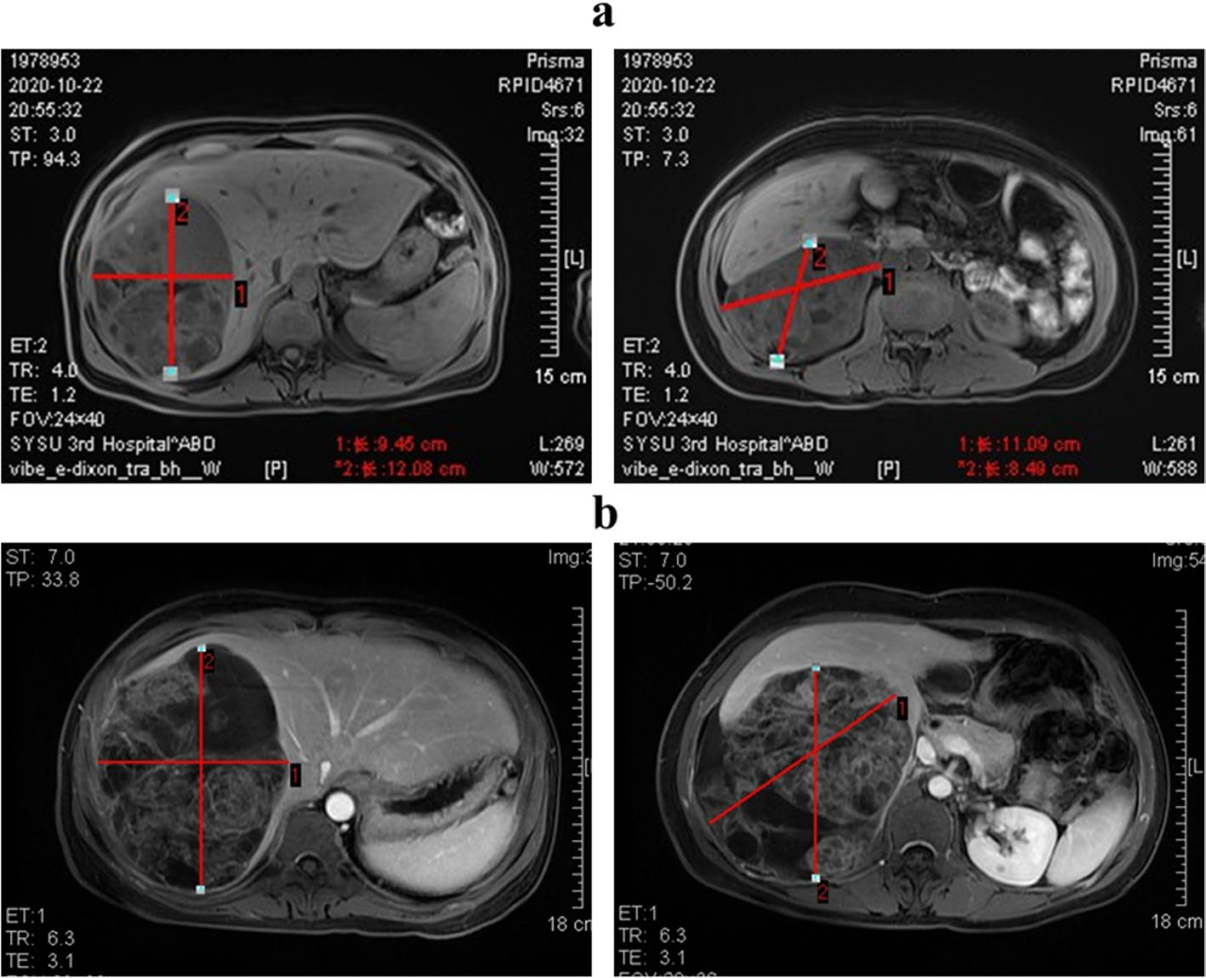
**a** MRI examination of the upper abdomen on October 22, 2020. **b** MRI examination of the upper abdomen on January 29, 2021 showed that the teratoma of the liver was alive and slightly bigger than before. Note: left picture represents the largest section of the mass in S VII-VIII of liver while the right one shows the largest section of the mass in S V-V

**Fig. 2:**
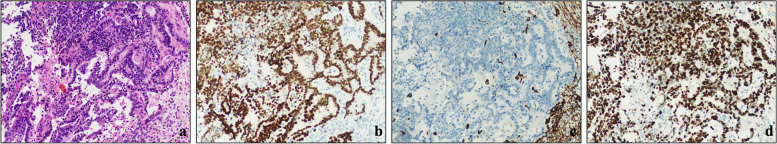
**a** Immature neuroectoderm-like structure was observed on pathological biopsy of the tumor (H&E, × 100). **b** Immunostaining of SALL-4 was positive. **c** Immunostaining of GFAP was negative. **d** Ki-67 index was extremely high (**b**-**d**, × 100). I of liver

A 44-year-old female presented with progressive abdominal distension especially after eating, accompanied by anorexia, fatigue and vomiting, was admitted to our hospital. MRI of the upper abdomen (Fig. 1a) revealed an irregular huge cystic-solid mass which located in both Segments V-VI and VII-VIII of the liver. The largest size of each section was approximately 12 cm × 9 cm (S VII-VIII) and 11 cm × 8 cm (S V-VI). The mass consisted of dense calcification, fat and soft tissue, poorly demarcated from the adjacent peritoneum, suggesting the possibility of malignant teratoma. No abnormalities were found during the examination of the uterus and bilateral adnexa. For serum tumor markers, carcinoma embryonic antigen (CEA) and β-human chorionic gonadotropin (β-HCG) levels were normal, but AFP level was elevated to 476 ng/ml (the normal range: 0–20 ng/ml). Then, biopsy specimens were obtained and immature neuroectodermal-like structure was observed (Fig. 2a), which showed positive staining for spalt like transcription factor 4 (SALL4, Clone#6E3, ZSGB-BIO) and negative for glial fibrillary acidic protein (GFAP, Clone#GA5, Vision BiosytemsTM) (Fig. 2b, c). Also, Ki-67 (Clone#MRX002, MXB Biotechnologies) staining showed high proliferative index (Fig. 2d). Therefore, the tumor was diagnosed as immature teratoma, which was not further graded because of the limited tissues.

After exclusion of chemotherapy contraindication, a systemic chemotherapy was delivered associating etoposide (75 mg/m^2^), with ifosfamide (1.2 g/m^2^) and cisplatin (20 mg/m^2^) each day, day 1 to day 4, every 3 weeks. After four cycles, MRI of the upper abdomen showed tumor survival and the size of tumor increased to 14 cm × 11 cm (S VII-VIII) and 13 cm × 12 cm (S V-VI) (Fig. 1b). However, the serum AFP level was nearly normal.

For further treatment, right hepatectomy, cholecystectomy and partial diaphragmectomy and diaphragmatic repair were performed. During the surgery, the tumor was found to protrude from the liver surface and adhere to the diaphragm, omentum, transverse colon, stomach and duodenum. The tumor size was about 28 cm × 17 cm × 15 cm, and it invaded the abdominal wall and part of the diaphragm. No metastatic tumor was detected in other parts of the abdominal cavity.

Macroscopically, the resected liver measured 30 cm × 24 cm × 13 cm. Cut sections of the liver revealed a solitary, round and cystic-solid mass with grayish or grey-yellow color, which sized 28 cm × 14 cm × 13 cm. The tumor was well-circumscribed but unencapsulated, adjacent to the liver capsule and about 2.5 cm distant from surgical margin.

**Fig. 3:**
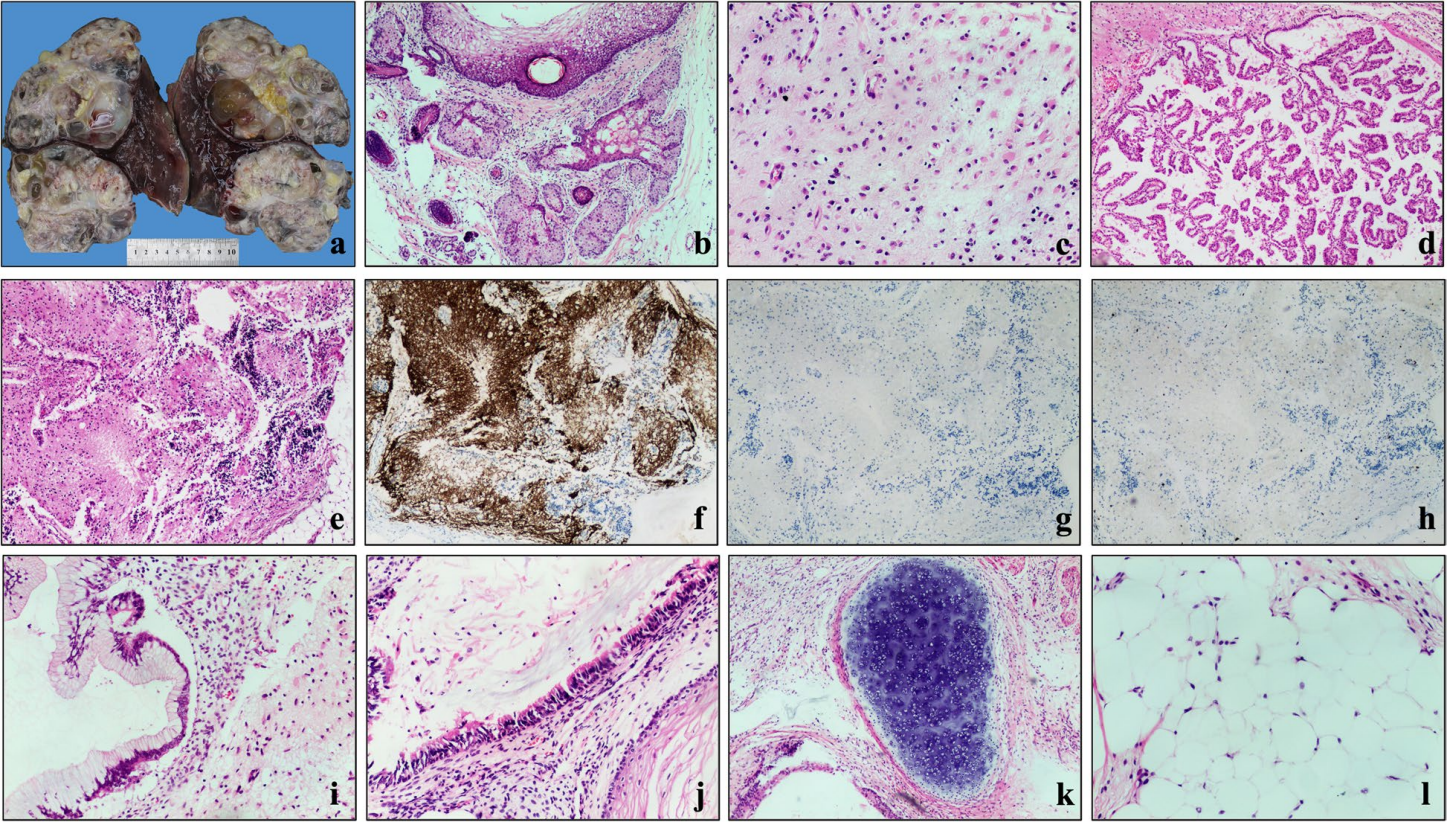
**a** The cut surface of the resected liver, illustrating a solitary, round and cystic-solid mass which included multiple cystic cavities, bone-like tissues, tender brain-like tissues, and lots of fat. Ectodermal derivatives including squamous epithelium, sebaceous glands, eccrine glands and hair follicle (**b**, H&E, × 40), and mature brain tissues such as glia and choroid could be seen (**c-d**, H&E, × 100). Some areas of dense glial cells (**e**, H&E, × 40) showed positive for GFAP(**f**) but negative for SALL-4(**g**), while Ki-67(**h**) index was low (**f**–**h**, IHC, × 40). **i-l** Intestinal epithelia (**i**), respiratory ciliated epithelium (**j**), mature cartilage (**k**), and adipose tissue (**l**) could also be observed (**i**, H&E, × 40; **j**-**l**, H&E, × 100)

The section was cystic and solid, showing multiple cystic cavities with different sizes (about 2–10 cm in diameter). The inner wall of the cyst was smooth, and the cavity was filled with jelly-like viscous liquid. For the solid areas, hard bone-like tissues, tender brain-like tissues, and multitudes of fat were visible (Fig. 3a).

On histologic examination, the tumor contained multiple mature types of tissues derived from three germ layers. Ectodermal derivatives including squamous epithelium, sebaceous glands, eccrine glands and hair follicle could be seen (Fig. 3b). Also, abundant mature brain tissues such as glia and choroid can be observed (Fig. 3c-d). In some areas, these cells were sheet arranged, with layers and polarities, but without significant cellular atypia and neural tube formation (Fig. 3e). On immunohistochemical staining, those densely cellular regions demonstrated positive for GFAP but negative for SALL-4, and Ki-67 index was low (Fig. 3f-h). Moreover, intestinal epithelia and respiratory ciliated epithelium (endoderm), mature cartilage and adipose tissue (mesoderm) could also be observed (Fig. 3i-l). The tumor was cut open along its long axis and extensively sampled, and no immature component was found. GTS was diagnosed finally.

**Fig. 4:**
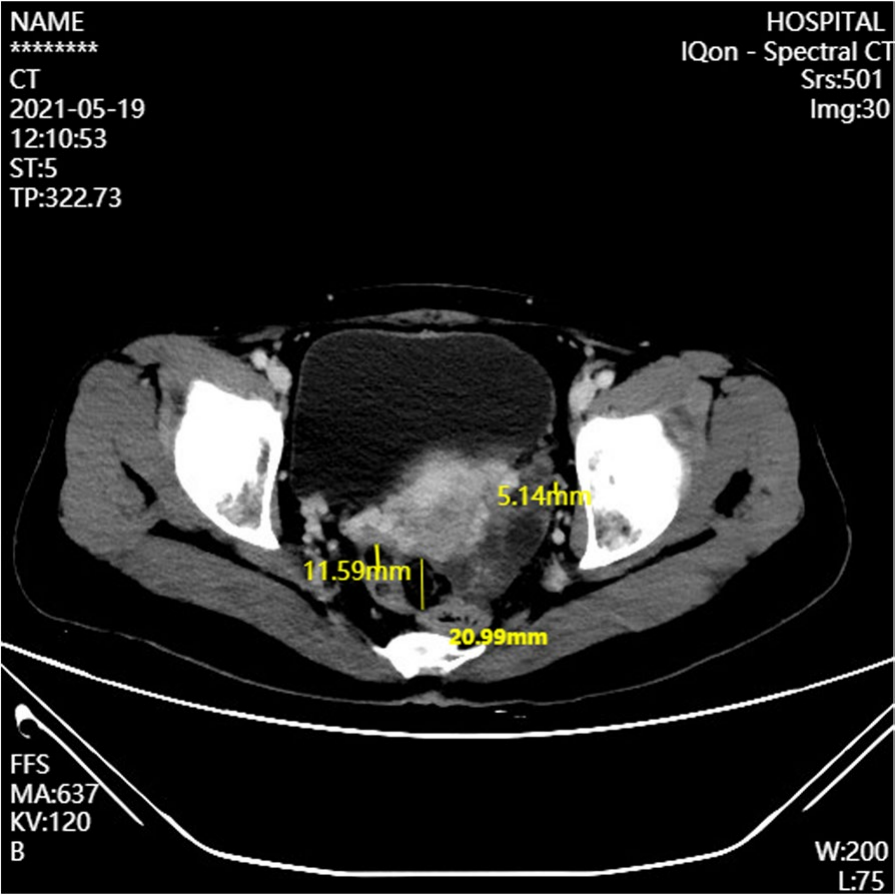
Pelvic CT revealed new-onset lesions in the appendages on both sides

**Fig. 5:**
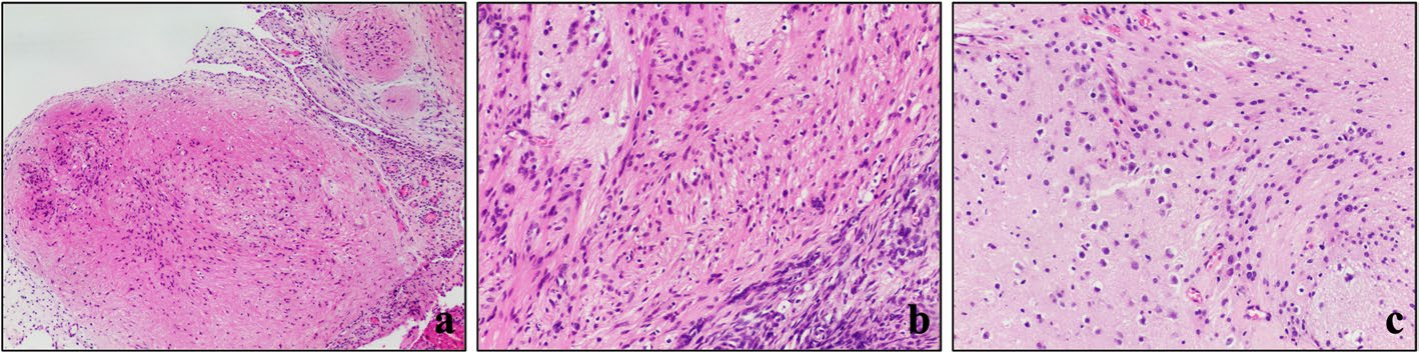
**a-c** A large amount of mature glial was observed (H&E; **a**, × 40; **b-c**, × 100)

Then the patient continued to receive 2 courses of chemotherapy, with the same type and dose of medications as before. However, after 2 months, a CT scan showed multiple small nodules in the bilateral adnexa, suggesting new-onset lesions (Fig. 4). Subsequently, the patient underwent the second surgery to resect total uterus and bilateral adnexa. Multiple nodules (0.5-2 cm in diameter) were found on the surface of the ovary, the front of the rectum, and the pelvis on both sides at surgery. Histologically, a large amount of mature glial was observed, while no immature components such as neural tube existed (Fig. 5a-c) with all tissues sampled. Based on clinical history, GP should be considered.

**Fig. 6:**
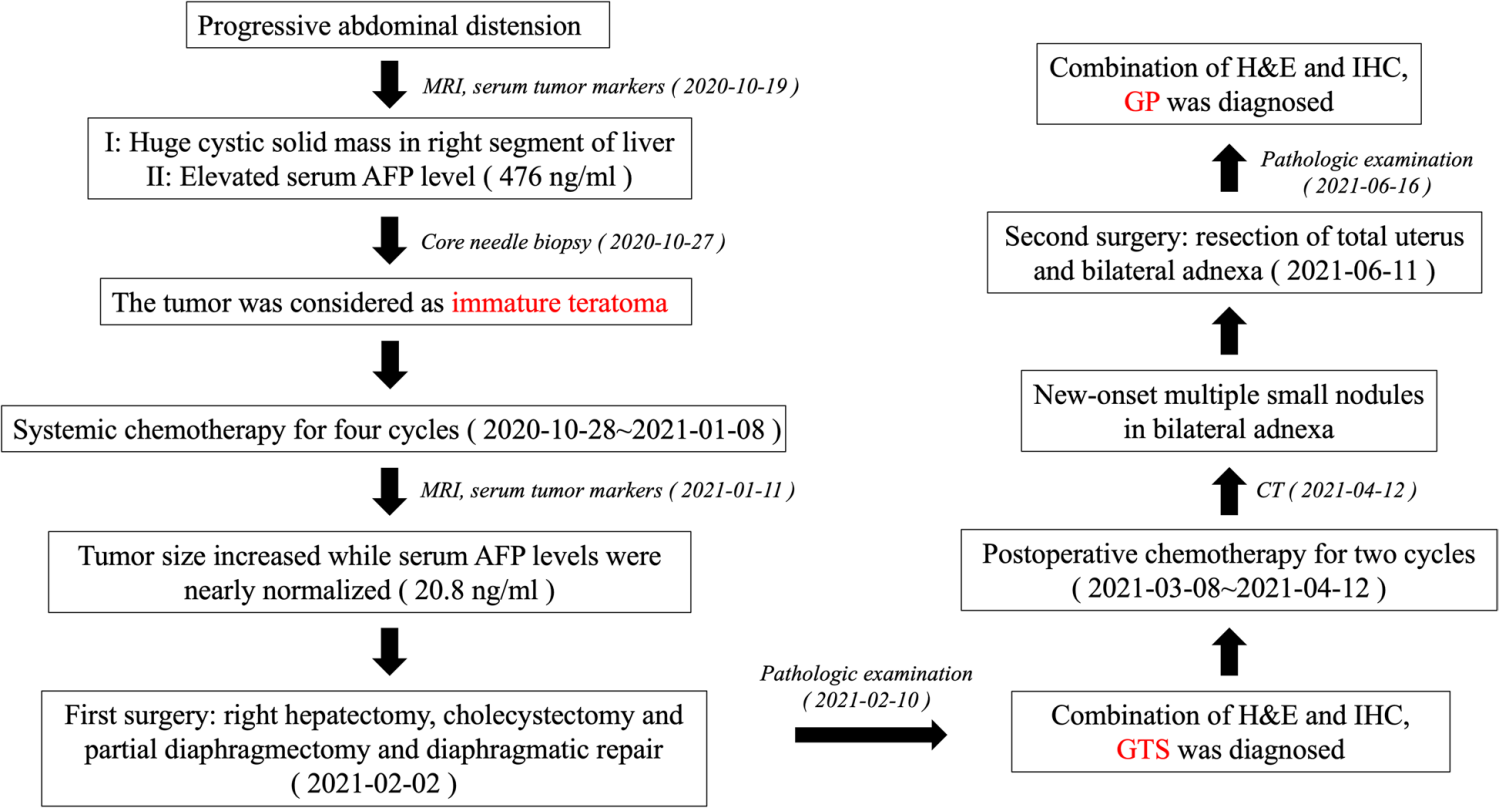
A brief flow chart of clinical diagnosis and treatment. H&E: Hematoxylin–Eosin staining. IHC: Immunochemistry. GTS: Growing Teratoma Syndrome. GP: Gliomatosis Peritonei. CT: Computed Tomography. MRI: Magnetic Resonance Imaging. AFP: Alpha Fetal Protein

**Fig. 7:**
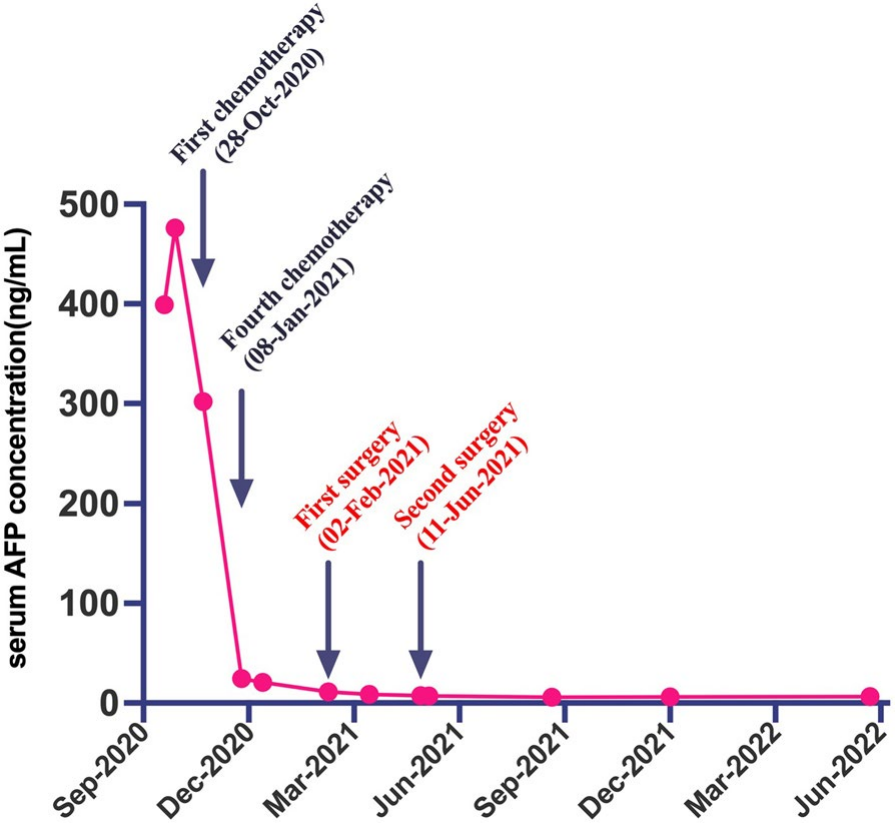
The serum AFP level of this patient continued to decrease with the progress of chemotherapy until it returned to the normal range. *First surgery: right hepatectomy, cholecystectomy and partial diaphragmectomy and diaphragmatic repair. ** Second surgery: resection of total uterus and bilateral appendage

A brief flow chart of clinical diagnosis and treatment was shown in Fig. 6. The patient was still in follow-up at the outpatient clinic and in good condition (Fig. 7).

## Discussion

In this study, we reported an extremely rare case of primary liver immature teratoma in adults. To date, only 5 cases, including the present one, have been reported in literature (Table 1). As shown in Table 1, there were 1 male and 4 females, and the median age was 22 years old. The largest tumor diameter varied from 16 to 42 cm. All cases were diagnosed as immature teratoma but only our case accompanied by both GTS and GP which is reported for the first time. Of the 5 patients, two died, two were cured, and our case remained in follow-up.

**Table 1:** Clinicopathological features of 5 patients with primary liver immature teratoma

*Case*	*Year*	*Age (gender)*	*Clinical symptoms*	*Tumor size*	*Pathology*	*Treatment*	*Prognosis*
1 [14]	2003	21 (Female)	Coughing, dyspnea, abdominal pain and fatigue	18 cm × 12 cm × 9 cm	Immature teratoma	Surgical resection and chemotherapy	Tumor recurrence and death
2 [15]	2010	34 (Male)	right upper quadrant abdominal pain	16 cm × 12 cm × 8 cm	Immature teratoma and yolk sac tumor	Surgical resection and chemotherapy	Tumor recurrence and death
3 [16]	2010	22 (Female)	right upper quadrant abdominal pain and sensation of fullness	27 cm in diameter	Immature teratoma	Surgical resection	Well during follow-up
4 [17]	2019	22 (Female)	abdominal pain, bloating, and diarrhea	31 cm × 25 cm × 42 cm	Immature teratoma with GTS	Surgical resection and chemotherapy	No signs of recurrence during 18 months of follow-up
5 (current case)	2021	44 (Female)	progressive abdominal distension	28 cm × 15 cm × 17 cm	Immature teratoma with GTS and GP	Surgical resection and chemotherapy	No signs of recurrence until 14-Jun-2022

In order to establish an unequivocal diagnosis of immature teratoma, 3 antibodies (SALL4, GFAP, Ki-67) were employed to distinguish primitive neuroepithelial tissues from mature counterparts. Immunohistochemical staining of Ki-67 was used to evaluate the tumor proliferation activity. GFAP is commonly considered as a marker for mature and differentiated astrocytes [24], which shows high immunoreactivity in mature glial cells but not in primitive neuroepithelial tissues of immature teratoma. As a sensitive and specific marker of germ cell tumors, SALL4 has been proved to be expressed in immature elements such as primitive neuroepithelial tissue and blastema-like stroma but not in mature tissues [25]. Thus, we selected a panel of these 3 antibodies to assist the diagnosis of immature teratoma. In terms of differential diagnosis, we first excluded metastasis of ovary teratoma, as CT/MRI did not show any mass in bilateral ovaries before the first chemotherapy. Moreover, a series of common malignant tumor of liver, including carcinosarcoma, hepatoblastoma, undifferentiated carcinoma should be considered. Unlike immature teratoma, both carcinosarcoma and undifferentiated carcinoma of liver do not contain mature epithelial or mesenchymal components such as squamous epithelium, mature cartilage or adipose tissue [26]. Hepatoblastoma occurs mainly in children but rarely in adults, consisting of variable combinations of epithelial and mesenchymal elements, which are absent in this case [27].

Malignant teratoma which continues to grow after chemotherapy may be suggestive of GTS. GTS is a rare clinical entity, occurring during or after systemic chemotherapy for immature teratoma or mixed malignant germ cell tumors, which presents as enlarged mass with normal serum tumor markers [8–11]. There are 3 main criteria to diagnose GTS [10, 28]. Firstly, the serum AFP and β-HCG levels should decrease or even return to normal after chemotherapy. Secondly, the tumor continued to grow after chemotherapy. Thirdly, pathological examination did not identify any immature tumor components. The serum AFP level of our case was continually decreased after chemotherapies and maintained at near normal level (Fig. 6), which fulfilled the first criterion. The tumor size of the case has slightly increased after 4 cycles of chemotherapy but no immature component was seen on histologic examination, which met the second and third criteria. So we finally got a diagnosis of GTS. The phenomenon described in the third criterion was formerly known as “chemotherapeutic retroconversion”, which was first proposed by DiSaia et al. [29]. Some studies suggested GTS and chemotherapeutic retroconversion may be the same phenomenon [8]. There were several hypotheses about the etiology of GTS. The most accepted one was that chemotherapy may selectively eliminate the immature component and allow benign components to further growth [30]. Another hypothesis is DNA-damaging chemotherapeutics could alter cell kinetics and then change cell behavior from malignance towards benignancy [31, 32]. Clinical courses of our case, to some extent, support the first hypothesis. However, more evidence should be explored to clarify the exact mechanism.

GP is defined as that mature neural tissues planted on the surface of the internal organs or the peritoneal wall. GP can occur in patients with mature and immature teratomas, while the planted elements are mature glial tissues. If the implants contain any immature components, GP should be excluded and metastasis of immature teratoma was considered [33]. In this case, new-onset tumors were detected in bilateral adnexa 2 months after the first teratoma resection of the liver, which was consistent with the characteristics of gliomatosis. Umekawa et al. suggested that GTS may be a part of the pathogenesis of GP since both GTS and GP are related to mature peritoneal glial implantation [33]. In our case, GTS occurred before GP. We supposed that as the tumor volume continued to increase after chemotherapy, it broke through the liver capsule, and part of the tumor spread to bilateral adnexal areas and the pelvic cavity. Imaging examinations including CT can only detect implants when a sufficient tumor bulk is present. Muller AM et al. [34] who reported two cases of GP and reviewed the literature, found that all 31 patients of ovary immaure terotoma were accompanied by GP following adjuvant chemotherapy conducted after the first operation. The authors concluded that the chemotherapeutic agent could induce somatic maturation of malignant cells. Thus, we can conclude that GP could also be considered as “chemotherapeutic retroconversion”. In general, the immature teratoma patients with no immature component found after chemotherapy showed excellent outcomes. Most cases are cured by complete resection [10]. If any immature components were detected in the post-chemotherapy tumor, traditional irradiation combined with chemotherapy may be ineffective. Immunotherapy, such as adoptive transfer of autogenous lymphokine-activated killer cells into immature tissues, seems promising [35].

Primordial germ cells follow a midline path and descend into the pelvis as ovarian and testicular cells, so they tend to appear in midline and paramedian locations [14]. Lim et al. pointed that mature teratoma may originate from fetal remnant implantation [36]. Base on this, some suggested that primordial germ cells migrating from the allantois hindgut in the first week of embryologic life may lead to immature teratoma by staying in midline extragonadal sites [20]. Another theory showed that the origin of some mature cystic teratoma is most likely pluripotential stem cell of corresponding organ or primordial germ cell before meiosis I [37], which may be the most possible hypothesis about the origination of primary liver teratoma. However, the pathogenesis of it still needs further study.

## Conclusions

In summary, we reported for the first time a rare case of primary liver immature teratoma with GTS and GP. Favourable clinical conditions were sustained at approximately 2 years of follow-up. Secondary GTS and GP may probably attributed to chemotherapeutic retroconversion. Because of its rarity, longer follow-up is needed to assess definitive efficacy and it’s a valuable supplement to the existing reports of relevant cases.

## Data Availability

All data generated or analysed during this study have been included in this published article.

## Abbreviations

GTS: Growing teratoma syndrome
GP: Gliomatosis peritonei
CT: Computed tomography
MRI: Magnetic resonance imaging
AFP: Alpha fetal protein
β-HCG: β-Human chorionic gonadotropin
CEA: Carcinoembryonic antigen
SALL4: Spalt like transcription factor 4
GFAP: Glial fibrillary acidic protein
